# Monodactyle Pigs

**Published:** 1898-02

**Authors:** 


Monodactyle Pigs.—Vasilescu relates the complete history of a family of one-toed pigs created and kept at the veterinary school of Bucharest. From numerous observations he draws the following conclusions:

1. The starting-point of the single-toed type in the pig has been a teratologic form—monstrosity.

2. This peculiarity, which is hereditary, has also been observed by Aristotle.

3. By careful selection and breeding among these single-toed forms, monodactylism has been transmitted from generation to generation, and, as experiments at Bucharest have indicated, can be reproduced indefinitely.—*Rev. Vét.*

